# Pulmonary hamartoma resembling multiple metastases: A case report

**DOI:** 10.3892/ol.2014.2043

**Published:** 2014-04-08

**Authors:** ZHENYA LU, FANGFANG QIAN, SHANWEN CHEN, GUOWEI YU

**Affiliations:** 1 Department of Internal Medicine, The First Affiliated Hospital, School of Medicine, Zhejiang University, Hangzhou, Zhejiang 310003, P.R. China; 2 The First Clinical Medicine, Wenzhou Medical University, Wenzhou, Zhejiang 325000, P.R. China; 3 Department of Urology, The First Affiliated Hospital, School of Medicine, Zhejiang University, Hangzhou, Zhejiang 310003, P.R. China; 4 Department of Thoracic Surgery, The First Affiliated Hospital, School of Medicine, Zhejiang University, Hangzhou, Zhejiang 310003, P.R. China

**Keywords:** pulmonary hamartoma, resemble, metastasis

## Abstract

The current study presents the case of a patient with multiple pulmonary nodules as observed by computed tomography. Furthermore, a marginal increase in fluorodeoxyglucose uptake was identified by positron emission tomography. Due to the appearance of multiple small nodules and a history of radical nephrectomy, a hypothetical diagnosis of pulmonary metastasis of a previously excised renal carcinoma was determined, which was confirmed by biopsy. Video-assisted thoracoscopic surgical resection of the nodules was proposed and pathological examination exhibited an unforeseen and rare observation.

## Introduction

Pulmonary metastasis is a common occurrence in patients with renal cancer and is usually treated with immunotherapy and novel agents that target angiogenesis (1). Certain clinical studies have indicated that the resection of pulmonary metastases (metastasectomy) may be a treatment option (1–2). However, the role of surgery for metastases originating from renal cancer has yet to be fully determined. Pulmonary nodules that appear in patients who have undergone nephrectomy for renal cancer are usually pulmonary metastases. However, the occurrence of metachronous lung tumors and certain benign diseases, particularly pulmonary hamartoma, are uncommon (3). The patient provided written informed consent.

## Case report

A 55-year-old female was referred to the First Affiliated Hospital, School of Medicine of Zhejiang University (Hangzhou, China) due to the presence of multiple round pulmonary nodules on a chest computed tomography (CT) scan, which was performed during a postoperative follow-up evaluation for renal cancer. The patient reported no history of cough, fever, chest pain, dyspnea, hemoptysis, weight loss or tuberculosis. In addition, no peripheral lymphadenopathy was detected and the routine blood test results, including a hemogram and renal and liver function tests, were within the normal ranges.

**Figure 1:**
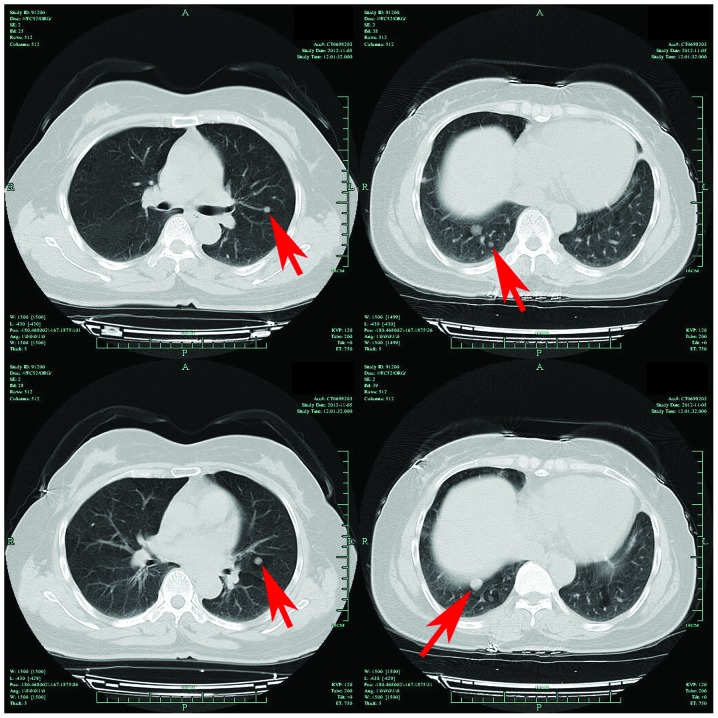
Contrast-enhanced computed tomography of the chest revealed multiple round pulmonary nodules, measuring ~10×12 mm with clear boundaries, in the lungs (indicated by the arrows).

**Figure 2:**
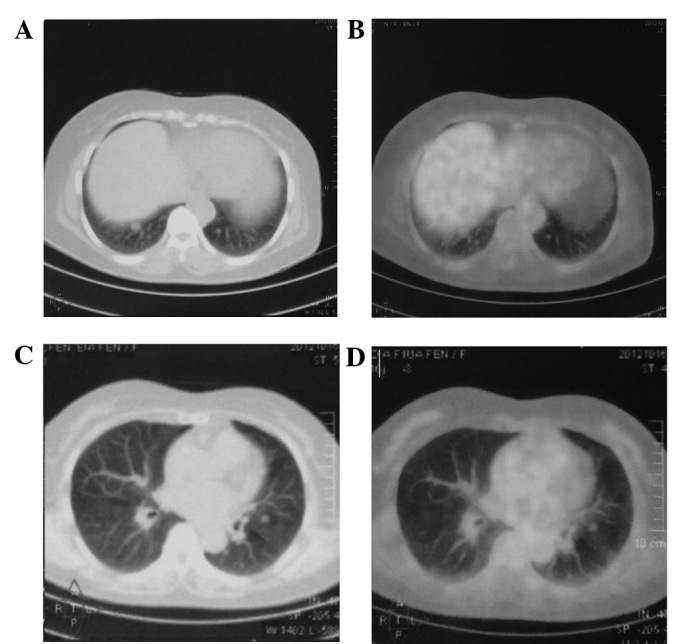
(A and C) Computed tomography (CT) and (B and D) ^18^F-fluoro-2-deoxy-D-glucose (FDG)-positron emission tomography/CT scan images exhibit a mild uptake of ^18^F-FDG in the pulmonary nodule indicative of malignancy.

The patient underwent a contrast-enhanced CT of the chest, which revealed multiple round pulmonary nodules measuring ~10×12 mm with clear boundaries in the lungs (Fig. 1). In addition, an ^18^F-fluoro-2-deoxy-D-glucose (FDG)-positron emission tomography (PET)/CT scan was performed to characterize the nodules, which exhibited a mild uptake of FDG that is indicative of malignancy (Fig. 2). In addition, bronchoscopy showed normal bronchi. Due to the presence of multiple small nodules with clear boundaries in the lungs and the history of a radical nephrectomy, a hypothetical diagnosis of pulmonary metastasis of a previously removed renal carcinoma was determined and subsequently confirmed by biopsy.

A video-assisted thoracoscopic nodulectomy was performed on the patient and frozen-section analysis revealed that the tumor was benign (possibly a pulmonary hamartoma) and the procedure was terminated.

**Figure 3:**
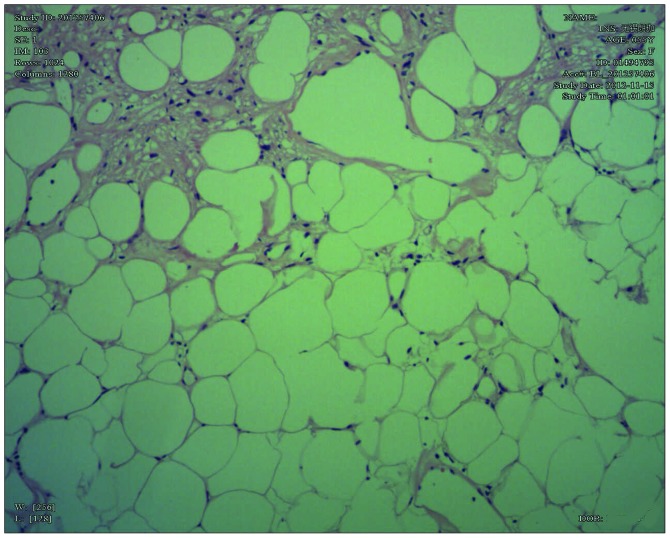
A section obtained from the pulmonary mass showed blood vessels, well-differentiated adipose tissue and polygonal cells. (stain, hematoxylin and eosin; magnification, ×100).

The anatomopathological examination revealed that the mass was a non-capsulated, regular lesion measuring 10×11×12 mm, with a firm and fibroelastic consistency. In addition, microscopic analysis revealed blood vessels, well-differentiated adipose tissue and polygonal cells (Fig. 3). Immunohistochemistry revealed pan-cytokeratin (−), melan-A (+), HMB45 (−), HHF35 (−), p53 (−), S-100 (+), desmin (−), cluster of differentiation 68 (−) and smooth muscle actin (+) expression, which is consistent with pulmonary hamartoma.

The patient recovered well and was discharged on the third postoperative day. After six months of postoperative follow-up, the patient has presented no signs of increasing multiple pulmonary nodules as assessed by computed tomography.

## Discussion

Pulmonary hamartoma account for 77% of all benign lung tumors and 4% of all solitary lung nodules (4–5). The lesion has been described as a benign neoplasm of the fibrous connective tissue of the bronchi surrounded by respiratory epithelium that commonly contains cartilage and adipose tissue, which does not comply with the usual histological distribution of the lung (6). In total, 90% of the hamartomas manifest as a solitary peripheral mass (5) and rarely occur in the form of multiple lesions (7). In addition, hartoma is more common in adults and the incidence rate is twice as high in males compared with females. The mean growth rate of hamartoma is 3.2±2.6 mm/year (8) and the occurrence of malignancy in hamartoma patients is possible. Certain studies have found that the incidence of bronchial carcinoma is 6.3-fold higher in patients with hamartoma than in a normal population, indicating the presence of an etiologic association (9). The appearance of pulmonary nodules during the follow-up evaluation of patients who have undergone nephrectomy is often confusing. Nine patients with a history of radical nephrectomy for renal cell carcinoma underwent the surgical removal of newly detected pulmonary nodules at the Hiroshima University Hospital (Hiroshima, Japan). Of these nine patients, six had metastatic lung tumors, two had bronchogenic primary carcinomas and one had a granulomatous infection (10).

The diagnostic algorithm in pulmonary hamartomas usually begins with structural imaging studies. Chest X-ray and CT are useful, however, magnetic resonance imaging has a limited role. Hamartomas are benign lesions containing normal pulmonary tissue and CT observations, such as internal fat or popcorn-like calcifications, are useful for distinguishing hamartomas from other malignancies (11–12). Certain studies have also demonstrated the presence of adipose tissue in 50% of the hamartomas that were evaluated by computed tomography (10). Radiological differentiation between benign and malignant nodules is determined according to size, margins, contour and internal characteristics, however, the interpretation may be fallacious (11–13). For example, in the present case, the lesion did not exhibit any such features on the CT scan. The CT also failed to reveal any signs of associated pulmonary tuberculosis.

FDG-PET scan is a useful non-invasive assessment in the differential diagnosis of indeterminate lung lesions, particularly in cases with an intermediate risk of malignancy (14). False-positive results from FDG-PET have been associated with focal infections or inflammatory conditions. In a previous study, six patients with pulmonary hamartoma underwent FDG-PET and only one demonstrated an accumulation of FDG (15–16). Tumors with a low metabolic rate, such as bronchioloalveolar carcinomas or carcinoids, may result in false-negative results, although, more recent results often describe mild FDG uptake in carcinoid lesions (17–18). Scott *et al* (19) also reported false-negative results in two patients with very small tumors. The rate of glycolysis in the tumor may have resulted in the low rate of FDG uptake and the actual amount of FDG uptake by the malignant tissue may have been relatively small, which resulted in a low overall FDG uptake. Furthermore, false-negative results are also possible in small tumors due to partial volume effects.

As the preoperative diagnosis of pulmonary hamartoma is often difficult, surgical resection is required for the differential diagnosis of lung cancer or metastatic lung tumors, unless clinical imaging reveals typical observations of pulmonary hamartoma. In the present study, considering the presence of multiple pulmonary nodules and the patient’s history of radical nephrectomy, metastasis was suggested as the initial diagnosis. Therefore, the histopathological diagnosis of hamartoma was unpredicted. The pulmonary nodules presented in patients who have undergone nephrectomy for renal cancer are not always pulmonary metastases and the confirmation of the histopathological diagnosis is fundamentally important to determine the optimal treatment method.

In conclusion. bronchoscopy with a biopsy is recommended for endobronchial lesions, as well as for patients with pulmonary symptoms, such as a cough, hemoptysis, recurrent pulmonary infections or atelectasis (20). In addition, percutaneous transthoracic aspiration biopsy diagnoses 85% of hamartomas, that present close to the thoracic wall, by differentiating them from nodules of other etiologies, such as renal cancer lung metastasis. Despite thorough clinical assessment with advanced imaging technology and needle biopsy, a number of patients continue to undergo surgery for benign disease. Therefore, future studies are required to identify novel strategies for the diagnosis and treatment of early-stage lung cancer (21). In cases where a diagnosis has not been determined due to the stiffness of the tumor, rendering a percutaneous biopsy useless, enucleation or resection via open thoracotomy or video-assisted resection is recommended (22).
